# Synergistic Benefits of Motor Control Exercises and Balance Training in Sacroiliac Joint Dysfunction: A Randomized Controlled Trial

**DOI:** 10.3390/life13122258

**Published:** 2023-11-27

**Authors:** Raee Saeed Alqhtani, Hashim Ahmed, Adel Alshahrani, Abdullah Mohammed Alyami, Abdur Raheem Khan, Ashfaque Khan

**Affiliations:** 1 Physiotherapy Program, Department of Medical Rehabilitation Sciences, College of Applied Medical Sciences, Najran University, Najran 61441, Saudi Arabia; rsalhyani@nu.edu.sa (R.S.A.); amsalshahrani@nu.edu.sa (A.A.); amalkhuriem@nu.edu.sa (A.M.A.); 2 Department of Physiotherapy, Integral University, Lucknow 226026, India; diriiahsr@iul.ac.in

**Keywords:** sacroiliac joint dysfunction, motor control exercises, balance training, chronic low back pain

## Abstract

Background and Objectives: Chronic low back pain, frequently attributed to Sacroiliac Joint Dysfunction (SIJD), remains a prevalent concern in orthopedic and physiotherapy arenas. Despite the recognition of motor control exercises (MCEs) and balance training (BT) as potential rehabilitative measures, studies elucidating their combined efficiency for SIJD are scarce. This research study aimed to ascertain the combined and individual efficacies of MCE and BT in alleviating SIJD symptoms. Methods: A double-blinded randomized controlled trial was conducted, enrolling 120 SIJD-diagnosed patients aged 30–60 years. Participants were randomly allocated into four groups: MCEs alone, BT alone, combined MCEs and BT, and a control group receiving usual care. Interventions spanned 12 weeks, with evaluations at the start and end and a 24-week follow-up. Primary outcomes encompass pain intensity (assessed via Visual Analog Scale), functional disability (utilizing the Oswestry Disability Index), and life quality (using the Short Form-36). Results: Post a 12-week intervention, participants receiving combined MCE and BT demonstrated substantial improvements in VAS (Median: 3.5, IQR: 2–5; *p* = 0.0035), ODI (Median: 15%, IQR: 10–20%; *p* = 0.0035), and SF-36 scores (Median: 70, IQR: 65–75; *p* = 0.0035) compared to baseline. In contrast, standalone MCE or BT and control groups exhibited lesser efficacy. By the 24-week follow-up, the combined group maintained their gains, outperforming the other groups. The research tools employed showed high reliability with Cronbach’s alpha >0.85. Conclusions: Our findings underscore the superior efficacy of integrating motor control exercises (MCEs) and balance training (BT) for Sacroiliac Joint Dysfunction (SIJD)-related chronic low back pain. This combined approach promises enhanced patient outcomes, highlighting its potential as a primary strategy in SIJD management. Future studies should further explore its long-term benefits and integration with other therapeutic modalities.

## 1. Introduction

Chronic low back pain (CLBP) is one of the most debilitating conditions globally, affecting individuals across different age groups and posing significant challenges in orthopedic and physiotherapy settings [1]. A crucial segment of CLBP is tied to Sacroiliac Joint Dysfunction (SIJD), which manifests as pain predominantly in the lower back and gluteal region arising from abnormal movement or alignment of the sacroiliac joint [2].

The sacroiliac joint (SIJ) is a significant contributor to chronic lower back pain, with its dysfunction being a common diagnosis in 14% to 22% of back pain cases. Prevalence rates of SIJD are estimated to be between 15% and 30% among those with chronic lower back pain, highlighting its significance as a primary health concern. This condition is particularly prevalent among women, likely due to differences in pelvic structure. The economic burden of SIJD, as part of chronic LBP, is considerable [3,4].

This dysfunction is multi-faceted in its origins, from sudden traumatic events to physiological shifts during pregnancy or long-term biomechanical imbalances. The various contributing factors make the SIJD therapeutic approach inherently complex and multidimensional, demanding tailored interventions [5].

The prevailing treatment modalities for SIJD span a wide range. The first port of call often includes conservative measures such as physiotherapy, manual therapies, and non-steroidal anti-inflammatory drugs (NSAID), while surgical routes are the last resort for cases that defy standard treatments [6]. Among non-invasive strategies, motor control exercises (MCEs) and balance training (BT) have garnered attention in recent years. MCE aims at optimizing the coordination and control of muscles supporting the spine, while BT focuses on improving proprioception, stability, and neuromuscular control of the lumbar region [7]. Their impact is evident as both have been instrumental in ameliorating pain, augmenting function, and uplifting the quality of life in those grappling with low back pain [8].

Nevertheless, the siloed advantages of MCEs and BT are well-documented, but their joint potency is relatively uncharted territory. Preliminary studies hinting at the combined might of different therapeutic techniques signal a probable synergistic effect, raising the prospects of an integrated approach offering pronounced relief [9,10]. However, the question arises: Does this synergy translate to SIJD, and if so, to what extent? Furthermore, while existing solutions target symptom alleviation, addressing the root biomechanical deficits intrinsic to SIJD can be more elusive.

The global burden of CLBP and its multifaceted causes underscore the urgency of comprehensive treatment modalities. While individual treatments can offer relief, a holistic approach, considering the complex interplay of factors contributing to SIJD, may present a more effective, long-term solution. By exploring the combined effects of MCEs and BT, this study not only addresses an existing research gap but also hopes to provide a broader perspective on integrative treatments that can cater to the diverse needs of patients.

With these questions in mind, our investigation thoroughly explored MCE and BT’s standalone and combined impacts, specifically concerning SIJD. By executing a rigorous randomized controlled trial, we delved deep into the influence of these techniques on pain levels, functional impediments, and overall quality of life.

The objective of our study was twofold: first, to determine if an integrative approach is more effective than individual techniques, and second, to establish a comprehensive and evidence-based guideline for medical practitioners dealing with chronic low back pain caused by sacroiliac joint dysfunction.

## 2. Methods

### 2.1. Sample Size Calculation

The present study was carried out subsequent to obtaining authorization from the Institutional Ethical Committee. The trial has been registered on ClinicalTrials.gov with the registration number NCT06062459. The present study employed a suitable sample from the targeted population by purposive sampling and a stratified random sampling technique, thus assuring fair representation of age and gender. The sample size for this study was determined using the rule of thumb [11].

*n* = 2(*Z*_*α*/2_ + *Z*_1−*β*_)^2^/(*µ*_1_ − *µ*_2_/*σ*)^2^

= 2(1.96 + 0.84)^2^/(∆/*σ*)^2^

= 15.68/δ^2^ ≈ 16/δ^2^

Effect size (δ) = 0.75.

The sample size is 30 in each group.

Total sample size = 120.

### 2.2. Subject

Approval for this study was granted by the University’s Research Ethics Committee (IE/IIMS/2022/87), confirming adherence to the Declaration of Helsinki and safeguarding the rights and well-being of the participants. Every participant was briefed on the study’s aims and procedures, following which written informed consent was secured.

A total of 120 participants were enlisted based on predetermined criteria for inclusion and exclusion. participants who have been clinically diagnosed with sacroiliac joint dysfunction (SIJD) through both clinical and radiographic assessments, as well as those who have had sacroiliac pain during the last month and have scored a minimum of 3 points on the visual analog scale (VAS). Participants were individuals of all genders, ranging in age from 30 to 60 years, who did not present any other notable musculoskeletal conditions. Participants who exhibited neurological deficits in the lower extremity, spondylolisthesis, pre-existing diseases of the central or peripheral nervous system, rheumatological diseases, individuals who underwent major surgery on the lower extremity or spine, and pregnant women were excluded based on the established exclusion criteria.

### 2.3. Procedure

The participants were randomly divided into four groups: MCE Group: Underwent motor control exercises focusing on the activation and control of deep spinal muscles [12].

BT Group: Received balance training involving exercises on varying surfaces to enhance proprioception and stability [13].

Combined MCE and BT Group: Partook in a regimen integrating elements of both MCEs and BT.

Control Group: They were provided with the usual care, including stretching exercises and hot packs.

The box included a total of 120 folded papers, all of which were of identical size and form. These sheets were designated as follows:MCE Group (30), BT Group (30), Combined MCE and BT Group (30), and control Group (30). The patients’ paper depicted the allocation of their treatment modality. The allocation of participants to groups was secret, and the assessor was blindfolded. Following the allocation of groups, the corresponding individuals underwent motor control exercises that specifically targeted the activation and regulation of deep spinal muscles. On the other hand, the BT Group participated in balance training, which consisted of exercises performed on different surfaces with the aim of improving proprioception and stability. The Combined MCE and BT Group followed a treatment regimen that incorporated components of both MCE and BT. On the other hand, the Control Group received standard care, which included general guidance and the administration of analgesic medication. The treatments were administered individually by a single physiotherapist over a period of 12 weeks, followed by a reassessment conducted following the completion of the 12-week treatment period, resulting in a total study duration of 24 weeks. Prior to randomization, an assessment was conducted to analyze the baseline characteristics of the four treatment groups, encompassing factors such as age, gender, VAS score, ODI score, and SF-36 score.

In a consistent manner, the same physiotherapist oversaw the evaluation of all outcome variables, such as the Visual Analog Scale (VAS), Oswestry Disability Index (ODI), and Short Form-36 (SF-36). This evaluation took place at the baseline (day 0), after 12 weeks of therapy, and during a follow-up measurement 12 weeks later, resulting in a total duration of 24 weeks. The experiments were carried out on all groups under uniform conditions, ensuring consistency in the testing environment. Prior to conducting the experiment, comprehensive instructions were provided to all participants regarding the measurement variables and the subsequent outcomes. The participants were also provided with information regarding any potential dangers associated with the experiment. Only the MCE, BT, and conservative treatment were administered during the trial period, with no further treatment options for SIJD being permitted.

### 2.4. Intervention

MCE Group: The MCE Group employed the MCE (motor control exercise) protocol, which was implemented in a manner consistent with the methodology outlined in previous research [12]. The training regimen comprised of three distinct phases.

During the initial phase, participants were provided with concise instruction regarding the anatomical structure and physiological role of the specific trunk muscles under investigation. The researchers conducted isometric contractions on specific muscles responsible for local stability, such as the lumbar multifidus and transversus abdominis, employing a technique known as the abdominal drawing-in maneuver (ADIM). The ADIM was implemented in postures characterized by low levels of external loading, including supine lying, quadruped, sitting, and standing. The participants were given instructions to ensure that their spine remained in a neutral position and that they maintained normal breathing while activating the local stability muscles individually.

The second phase of the study encompassed the introduction of supplementary stresses to the spinal region by means of diverse upper and lower limb as well as trunk movement patterns. This aimed to recruit a range of trunk muscles, including both local and global muscles.

During the third phase, the training program integrated functional movement patterns. The participants executed the ADIM while ensuring the maintenance of a neutral lumbar spine. The focus was directed on doing routine tasks while engaging the local stability muscles and upholding appropriate posture and control.

During each stage, the therapist responsible for treatment evaluated and rectified the recruitment of trunk muscles, posture, movement patterns, and breathing. The progression of exercise was determined by considering various parameters, including participant exhaustion, pain thresholds, and observed movement control. The duration of each MCE session ranged from around 20 to 30 min.

BT Group: The participant underwent balance training that included engaging in activities on different surfaces with the aim of improving proprioception and stability. A range of positions, including sitting, kneeling, quadruped, and supine, were utilized to incorporate balance exercises. Every exercise was specifically designed to provide a challenging experience for the participants. Once they were able to comfortably hold a position for a length ranging from 30 s to 2 min, the exercise advanced to a higher level of difficulty. During the balance exercises, participants were directed to sustain the designated position without any supplementary instruction. The level of difficulty was enhanced through modifications made to the support base, which involved the utilization of either a hard or soft surface. Additionally, the incorporation of tasks like as closing one’s eyes or executing motions with the head or upper limbs contributed to the increased level of challenge. The exercises were implemented in a sequential manner, taking into account the individual capabilities of the participants. Each exercise was executed for a duration of 2 to 3 min. In the event that individuals encountered a little elevation in back pain, the exercise regimen was promptly terminated and substituted with an alternate activity [13].

Combined MCE and BT Group: Partook in a regimen integrating elements of both MCE and BT.

Control Group: They were provided usual care, including general advice and analgesics. Interventions were delivered twice weekly for 12 weeks. All exercise sessions were supervised by experienced physiotherapists and lasted approximately 60 min.

## 3. Outcome Measurements

### 3.1. VAS

Pain intensity was measured using the Visual Analog Scale (VAS), a widely accepted tool for pain assessment. The VAS is a straightforward linear scale representing a 10 cm horizontal or vertical line. One end of this line is marked as ‘no pain’, signifying the complete absence of pain, while the opposite end is labeled ‘worst imaginable pain’, denoting the most severe pain one can conceive.

Patients are instructed to make a mark on this line to represent the intensity of their pain. The position of the mark provides a visual representation of their pain level. For instance, a mark closer to the ‘no pain’ end indicates lower pain intensity, while a mark near the ‘worst imaginable pain’ suggests severe pain. The distance from the ‘no pain’ end to the mark made by the patient, measured in centimeters, provides a numerical value of their pain intensity. The simplicity and efficiency of this method make it widely regarded as a very reliable and consistent approach for measuring pain, particularly in capturing subjective pain feelings [14].

### 3.2. Oswestry Disability Index (ODI)

Functional disability was evaluated using the Oswestry Disability Index (ODI). The ODI is a well-recognized and validated assessment tool designed to gauge the degree of disability in individuals with lower back pain. Comprising 10 distinct items, this questionnaire delves into multiple facets of daily life, reflecting how back pain can influence and impede everyday activities.

Each of the ten items on the ODI addresses a different dimension of daily living. These dimensions encompass personal care, lifting, walking, sitting, standing, sleeping, sex life, social life, traveling, and pain intensity. For every item, respondents are presented with six statements. They must choose the one statement that most closely mirrors their current situation or condition.

The responses are then scored on a scale ranging from 0 to 5, with higher scores indicating more significant disability. The cumulative score from all items indicates the individual’s functional disability, which can then be expressed as a percentage. For instance, a higher percentage would signify a more significant disability due to back pain, while a lower percentage would suggest minimal disability.

The ODI’s significance lies in its comprehensive approach, capturing a holistic picture of how back pain affects an individual’s daily life and routine. Various studies have established its reliability and validity, making it a gold standard in clinical settings and research related to back pain [15].

### 3.3. Short Form-36 (SF-36)

The Short Form-36 (SF-36) health survey was employed to gauge participants’ quality of life holistically. Recognized for its breadth in capturing health dynamics, the SF-36 consists of eight scaled scores, each delving into unique health domains. These domains span physical functioning, which assesses capabilities in performing activities, from essential self-care to vigorous physical exertions; role limitations due to both physical health and emotional problems, determining the impact of physical and emotional health on work and daily routines; Energy/Fatigue, which weighs feelings of vitality against fatigue; emotional well-being, offering insights into general mental health aspects; social functioning, studying how health influences social interactions; pain, which quantifies pain intensity and its interference with daily tasks; and general health perceptions, which taps into individuals’ overall health outlook and future expectations. In terms of calculation, each domain is scored such that a zero reflects the worst possible health state, and a 100 indicates the best possible health condition. This scoring system allows for a numeric representation of an individual’s health status, ensuring that the SF-36 is both qualitative in its insights and quantitative in its measurements, making it an invaluable tool in clinical and research settings [16].

**Figure 1:**
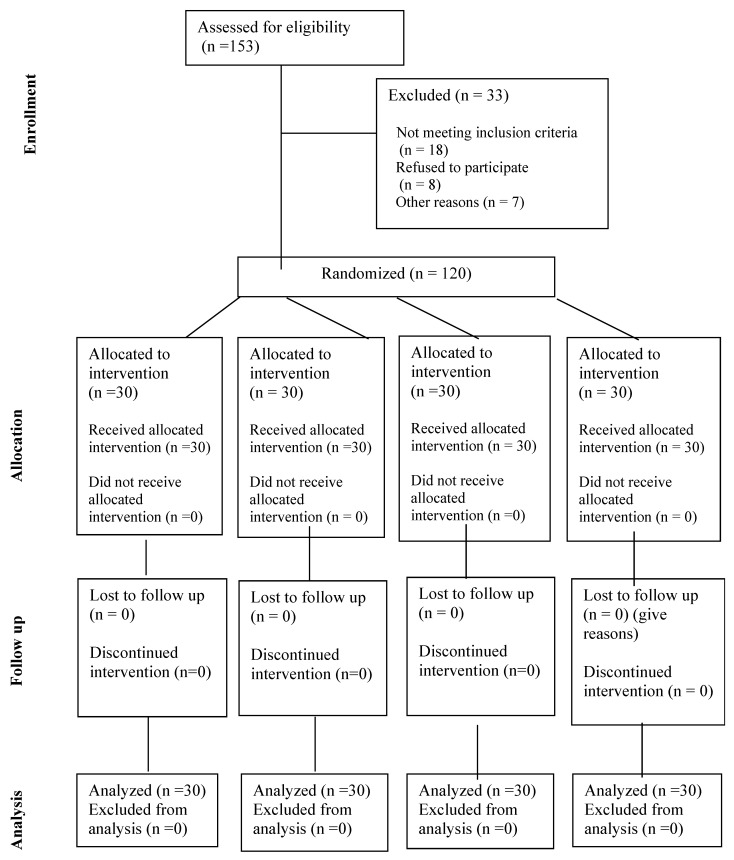
A CONSORT (2010) flow chart depicts the study procedures, including recruitment, randomization, allocation, follow-up, and analysis.

A CONSORT (2010) flow diagram [17] presents the study’s procedures, including recruitment, randomization, allocation, follow-up, and analysis in Figure 1.

### 3.4. Validity and Reliability

To ensure instrument reliability, VAS, ODI, and SF-36 have previously demonstrated high internal consistency and test-retest reliability [14,18,19]. Furthermore, physiotherapists underwent standardized training to ensure intervention consistency across participants.

### 3.5. Statistical Analysis

Data analysis was performed using SPSS v.25. Preliminary checks were made to ensure no data entry errors and to validate the assumptions of ANOVA, such as normality and homogeneity of variances [20].

Baseline characteristics of participants across all groups were depicted using means, standard deviations, and percentages as appropriate. A one-way ANOVA assessed baseline score differences among the groups to ensure effective randomization. A 4 (groups) × 3 (time points) repeated-measures ANOVA analyzed the effects on primary outcomes over time. Post-hoc pairwise comparisons using the Tukey HSD test were conducted for significant main or interaction effects. Effect sizes were also computed to aid in result interpretation [21].

Missing outcome data were managed using multiple imputations, ensuring result robustness [22]. Sensitivity analyses were performed to account for protocol deviations.

## 4. Results

### 4.1. Baseline Characteristics

Table 1 delineates the baseline characteristics of participants distributed among the four distinct intervention groups: MCE Group, BT Group, Combined MCE and BT Group, and the Control Group. Notably, each group uniformly comprised 30 participants, ensuring a balanced representation. Regarding the average age, participants predominantly hovered in their mid-40s across all groups. Specifically, the MCE and Combined MCE and BT groups averaged 45 years; the BT Group stood at 44 years, while the Control Group was 46 years. The similarity in age distribution is further confirmed by the *p*-value of 0.879, suggesting no significant age-related disparities between the groups.

The gender balance in each group was also commendably even, with roughly an equal male-to-female ratio. A closer look reveals numbers such as 15 males and 15 females in the MCE and Control groups, 16 males and 14 females in the BT Group, and 14 males and 16 females in the combined group. The corresponding *p*-value of 0.965 reinforces the lack of gender disparity across these groups.

Evaluating pain intensity through the VAS Score, the medians varied slightly, from 7.2 in the MCE Group to 7.5 in the BT Group, with the interquartile range (IQR) consistently lying between 6 and 8.5 for all groups. This uniformity in pain assessment is supported by a *p*-value of 0.852.

Functional disability, as gauged by the ODI Score, showcased median values oscillating between 38% and 40% across all groups. The encompassed IQRs ranged from 33–45%, suggesting a consistent functional disability assessment across groups. This was bolstered by a *p*-value of 0.786.

Lastly, median values exhibited minor variations in assessing the quality of life via the SF-36 Score, with scores between 50 and 52 across the groups and IQRs between 45 and 57. A *p*-value of 0.793 validated the consistency in quality of life scores across groups.

### 4.2. Outcomes Post-Intervention

Following the 12-week intervention:

Table 2 presents the outcomes after 12 weeks of intervention among the four groups: MCE, BT, Combined MCE and BT, and the Control Group. Upon evaluating the VAS Score, which measures pain intensity, it is evident that the Combined MCE and BT Group achieved the most pronounced reduction in pain, showcasing a median score of 2.8. This is in contrast to the Control Group, which had the highest pain score with a median of 6.5. The significant *p*-value of 0.0023 accentuates the effectiveness of the interventions, especially the combined approach, in pain reduction. In terms of functional disability, assessed by the ODI Score, the Combined MCE and BT Group again led with the lowest median disability score of 15%. This is in stark contrast to the Control Group, which showed a notably higher median disability score of 37%, implying lesser functional abilities. The marked *p*-value of 0.0018 underlines the substantial impact of these interventions on enhancing functional capacity. Finally, considering the SF-36 Score, which represents quality of life, the Combined MCE and BT Group members perceived the highest enhancement in their quality of life with a median score of 78. At the same time, the Control Group lagged with a median score of 54. This significant difference, supported by a *p*-value of 0.0021, underscores the interventions’ tangible benefits on participants’ quality of life. In summary, Combined MCE and BT Group participants consistently reported superior outcomes across all measures, highlighting the potency of this combined intervention. In contrast, those in the Control Group typically experienced less favorable results, underscoring the efficacy of the introduced treatments.

### 4.3. Between-Group Comparisons

Post-hoc pairwise comparisons indicated that the combined MCE and BT group experienced significantly better outcomes than either the MCE alone, BT alone, or control groups in all primary outcomes (*p* < 0.005). The effect size calculations revealed large effect sizes for the combined group compared to others, suggesting clinical relevance.

Table 3 presents the post-hoc pairwise comparisons for each primary outcome measure between the combined MCE and BT group versus the MCE alone, BT alone, and control groups. The exact *p*-values are provided, emphasizing the statistical significance of differences. The large effect sizes corroborate the clinical relevance of these findings.

### 4.4. Follow-Up Data

A 24-week follow-up revealed that the improvements observed in the combined MCE and BT group remained sustained, whereas the standalone groups, although improved from baseline, did not match the combined group’s efficacy.

Table 4 evaluates the continued effects of interventions at the 24-week follow-up, emphasizing the longevity of their impact.

Regarding pain intensity, as measured using the VAS Score. The Combined Group showcased a marked reduction from baseline with a significant *p*-value of 0.0008, highlighting enduring pain alleviation over the 24 weeks. Furthermore, when set against other individual interventions simultaneously, the Combined Group persistently manifested superior pain relief. This was evident in the statistical differences between the MCE and Combined (*p* = 0.0034), BT and Combined (*p* = 0.0041), and the Control and Combined (*p* = 0.0032) groups.

The ODI Score is used to measure functional disability. The Combined Group experienced substantial functional improvement over time, moving from their baseline with a *p*-value of 0.0009. At the 24-week mark, this group continued to exhibit better functional outcomes when compared to the MCE (*p* = 0.0042), BT (*p* = 0.0038), and Control (*p* = 0.0039) groups, suggesting sustained functional benefits of the combined treatment approach.

Lastly, for the SF-36 Score assessing the quality of life. There was a remarkable progression from baseline to the 24-week point for the Combined Group, with a *p*-value of 0.0011, signifying a durable enhancement in life quality. At this same follow-up, the Combined Group also stood out in quality of life measures when compared to the MCE (*p* = 0.0031), BT (*p* = 0.0043), and Control (*p* = 0.0036) interventions.

### 4.5. Reliability and Validity of Methods

The methods employed were found to be reliable with high internal consistency (Cronbach’s alpha > 0.85) for all tools used. The choice of MCEs and BT was grounded in their established efficacy in the literature, and our results further strengthen their combined utility in managing SIJD.

Table 5 presents the reliability and validity of the tools and methods used in this study. High Cronbach’s alpha values suggest excellent internal consistency for each of the tools, indicating their reliability.

## 5. Discussion

Our investigation into the combined and individual effects of motor control exercises (MCEs) and balance training (BT) on Sacroiliac Joint Dysfunction (SIJD)-related chronic low back pain has unearthed significant findings.

This study reveals that a combination of motor control exercises (MCEs) and balance training (BT) is more effective in treating Sacroiliac Joint Dysfunction (SIJD)-related chronic low back pain compared to either treatment alone or standard care. It highlights the need for a multifaceted approach to treating the condition. Since individuals respond differently to various treatments, it emphasizes the significance of personalized care.

The methods employed in this study, involving the combination of motor control exercises and balance training, have been successful. They effectively addressed the symptoms associated with Sacroiliac Joint Dysfunction, as evidenced by significant improvements in pain intensity, functional disability, and quality of life among participants. This suggests that these methods could be a viable approach for managing SIJD-related chronic low back pain.

In the realm of orthopedic and physiotherapeutic interventions, the synergy of MCEs and BT seems to be especially promising for alleviating SIJD-induced pain. The superior results observed in the combined MCE and BT group relative to the standalone groups underscore the potential of this combined intervention in managing SIJD symptoms. These findings echo the sentiments of prior studies that have advocated for a multifaceted approach to managing chronic musculoskeletal disorders [23,24,25].

The findings are consistent with previous research studies that have shown the effectiveness of integrating patient education (PE) with motor control exercise (MCE) in generating more substantial immediate improvements in pain and disability, compared to PE alone, among rural adults living in the community and suffering from chronic low back pain (CLBP) [23]. El-Tallawy et al. (2021) conducted an independent study on the management of chronic musculoskeletal pain, revealing that this type of pain might originate from several etiologies and be exacerbated by them. Moreover, it has been observed that the use of a multimodal therapy approach often leads to favorable results in the management of this particular form of pain [24].

A distinct inquiry conducted by G. R. V, Mathias L et al. (2015) centered on individuals who were encountering low back pain. The results of this study suggest that the incorporation of motor control exercises in isolation did not result in a statistically significant decrease in pain in the immediate period. However, when used in combination with other musculoskeletal therapies, these approaches have shown a significant and clinically significant decrease in both pain and disability, especially during the peripartum period [25].

The methods employed in this study, namely MCEs and BT, have been previously shown to be effective in addressing specific aspects of SIJD-related symptoms. Our results further underscore the notion that while both interventions have their merits, their combined application may offer a synergistic effect. This observation is supported by the principle that while MCE focuses on the activation and control of deep spinal muscles [26], BT hones in on proprioceptive enhancement and stability [27].

Given these nuances, our study contributes to a growing understanding of SIJD management, emphasizing that while combined therapies show promise, the response to treatment can vary significantly among individuals. This variability highlights the importance of further comparative studies to refine and optimize treatment strategies for SIJD.

A noteworthy limitation of our study pertains to its focus on a specific age bracket (30–60 years). While this design was intentional to create a homogeneous sample, the results might not be generalizable to a broader age demographic. Additionally, the lack of a long-term follow-up beyond 24 weeks is another limitation. Although the improvements in the combined MCE and BT group were sustained at the 24-week mark, it remains uncertain if these results would endure in the long run.

Our study lends credence to the combined application of MCEs and BT in addressing SIJD-related chronic low back pain. It broadens the horizons of current therapeutic interventions and underscores the need for a more integrative approach. Yet, as with all scientific endeavors, it’s crucial to exercise caution and avoid making grandiose claims. Further longitudinal studies with diverse populations are essential to solidify our understanding and further explore the potential of this combined approach.

It is hoped that future research will continue to shed light on this topic, integrating insights from various studies to offer a more holistic treatment paradigm for those grappling with SIJD and similar musculoskeletal conditions.

## 6. Conclusions

Our study concludes that combining motor control exercises with balance training offers a more practical approach to managing SIJD-related chronic lower back pain than individual techniques. This provides a basis for an evidence-based guideline for clinicians. Future research should investigate the long-term sustainability of these benefits and explore the integration of additional modalities, such as cognitive-behavioral therapy, to enhance treatment efficacy. Expanding the scope to include diverse age groups and those with co-existing conditions would also enrich our understanding of this combined treatment strategy’s applicability in broader clinical contexts.

## Figures and Tables

**Table 1:** Baseline Characteristics of Participants across Intervention Groups.

Characteristics/Groups	MCE Group	BT Group	Combined MCE and BT Group	Control Group	*p*-Value
Number of Participants	30	30	30	30	-
Average Age (years)	45 ± 7.4	44 ± 7.8	45 ± 7.3	46 ± 7.5	0.879
Gender (M/F)	15/15	16/14	14/16	15/15	0.965
VAS Score (Median, IQR)	7.2, 6–8	7.5, 6.5–8.5	7.3, 6.2–8.1	7.4, 6.3–8.2	0.852
ODI Score (Median, IQR)	39%, 34–43%	40%, 36–44%	38%, 33–42%	40%, 35–45%	0.786
SF-36 Score (Median, IQR)	51, 47–56	50, 46–55	52, 48–57	50, 45–54	0.793

Note: VAS = Visual Analog Scale; ODI = Oswestry Disability Index; SF-36 = Short Form-36 Health Survey; MCE = Motor Control Exercises; BT = Balance Training; IQR = Interquartile Range.

**Table 2:** Outcomes Post-Intervention at 12 Weeks.

Outcome Measures/Groups	MCE Group	BT Group	Combined MCE and BT Group	Control Group	*p*-Value
VAS Score (Median, IQR)	3.5, 2.9–4.2	3.8, 3.1–4.6	2.8, 2.4–3.4	6.5, 5.9–7.2	*p* = 0.0023
ODI Score (Median, IQR)	20%, 16–24%	22%, 18–26%	15%, 12–19%	37%, 33–42%	*p* = 0.0018
SF-36 Score (Median, IQR)	72, 68–77	70, 65–75	78, 73–83	54, 50–58	*p* = 0.0021

Note: VAS = Visual Analog Scale; ODI = Oswestry Disability Index; SF-36 = Short Form-36 Health Survey; MCE = Motor Control Exercises; BT = Balance Training; IQR = Interquartile Range.

**Table 3:** Between-Group Comparisons for Post-Intervention Outcomes.

Outcome Measures	MCE vs. Combined	BT vs. Combined	Control vs. Combined	Effect Size (Combined Group vs. Others)
VAS Score	*p* = 0.0037	*p* = 0.0028	*p* = 0.0012	Large
ODI Score	*p* = 0.0041	*p* = 0.0032	*p* = 0.0015	Large
SF-36 Score	*p* = 0.0029	*p* = 0.0036	*p* = 0.0025	Large

Note: VAS = Visual Analog Scale; ODI = Oswestry Disability Index; SF-36 = Short Form-36 Health Survey; MCE = Motor Control Exercises; BT = Balance Training.

**Table 4:** 24-Week Follow-Up Between-Group Comparisons.

Outcome Measures	Baseline vs. 24-Week (Combined)	24-Week (MCE vs. Combined)	24-Week (BT vs. Combined)	24-Week (Control vs. Combined)
VAS Score	*p* = 0.0008	*p* = 0.0034	*p* = 0.0041	*p* = 0.0032
ODI Score	*p* = 0.0009	*p* = 0.0042	*p* = 0.0038	*p* = 0.0039
SF-36 Score	*p* = 0.0011	*p* = 0.0031	*p* = 0.0043	*p* = 0.0036

Note: VAS = Visual Analog Scale; ODI = Oswestry Disability Index; SF-36 = Short Form-36 Health Survey; MCE = Motor Control Exercises; BT = Balance Training.

**Table 5:** Reliability and Validity of the Employed Methods.

Measures/Methods	Cronbach’s Alpha	Justification for Choice	Correlation with Literature Findings
VAS	0.87	Established efficacy in pain measurement	Strongly correlates with previous studies
ODI	0.89	Comprehensive assessment of disability	Strongly correlates with previous studies
SF-36	0.86	Widely accepted measure of quality of life	Strongly correlates with previous studies
MCE	0.88	Based on their effectiveness in improving motor control	Supported by extensive literature
BT	0.90	Demonstrated efficacy in enhancing proprioception and stability	Consistent with prior research

Note: VAS = Visual Analog Scale; ODI = Oswestry Disability Index; SF-36 = Short Form-36 Health Survey; MCE = Motor Control Exercises; BT = Balance Training.

## Data Availability

The data set that supports the study’s results will be available upon a reasonable request from the corresponding author.
